# Advanced nanoparticles for osteoarthritis: a review

**DOI:** 10.3389/fmed.2025.1651659

**Published:** 2025-10-10

**Authors:** Kang Yang, Yong Yuan, Li-Min Guo, Wen-Dong Luo, Chong-Hua Dai, Feng-Xiang Xia, Feng Lin, Xing-Xiong Jiang

**Affiliations:** ^1^Department of Traumatology, The Second Affiliated Hospital of Kunming Medical University, Kunming, Yunnan, China; ^2^Department of Orthopedics, The First People's Hospital of Luliang County, Qujing, China; ^3^Department of Orthopedics, The First People's Hospital of Xuanwei City, Qujing, China

**Keywords:** OA, PLGA, nanoparticle, drug, cartilage

## Abstract

Osteoarthritis (OA) is the most common chronic joint disease, characterized by whole-joint degenerative disease with cartilage degeneration as the primary pathogenesis. It is also a major cause of disability and increased social costs, particularly among the elderly. With the aging population and increasing obesity rates, the incidence of OA increases annually. The main symptoms include joint pain and loss of joint function, which severely impact the quality of life and daily activities of patients. Despite numerous treatments attempted over the past few decades, the long-term treatments have been disappointing. The main challenge lies in the very low bioavailability of drugs within the joint cavity, Therefore, the development of a therapeutic approach with cartilage targeting and efficient bioavailability is the key point to address OA. This paper summarizes the latest research on the use of PLGA in drug delivery for the treatment of OA, which provides an important foundation and a more comprehensive perspective for the subsequent drug treatment of joint diseases. We hope this will lead to more accurate and effective treatment plans for arthritis patients and promote the continuous advancement of the medical field.

## 1 Introduction

**Figure 1:**
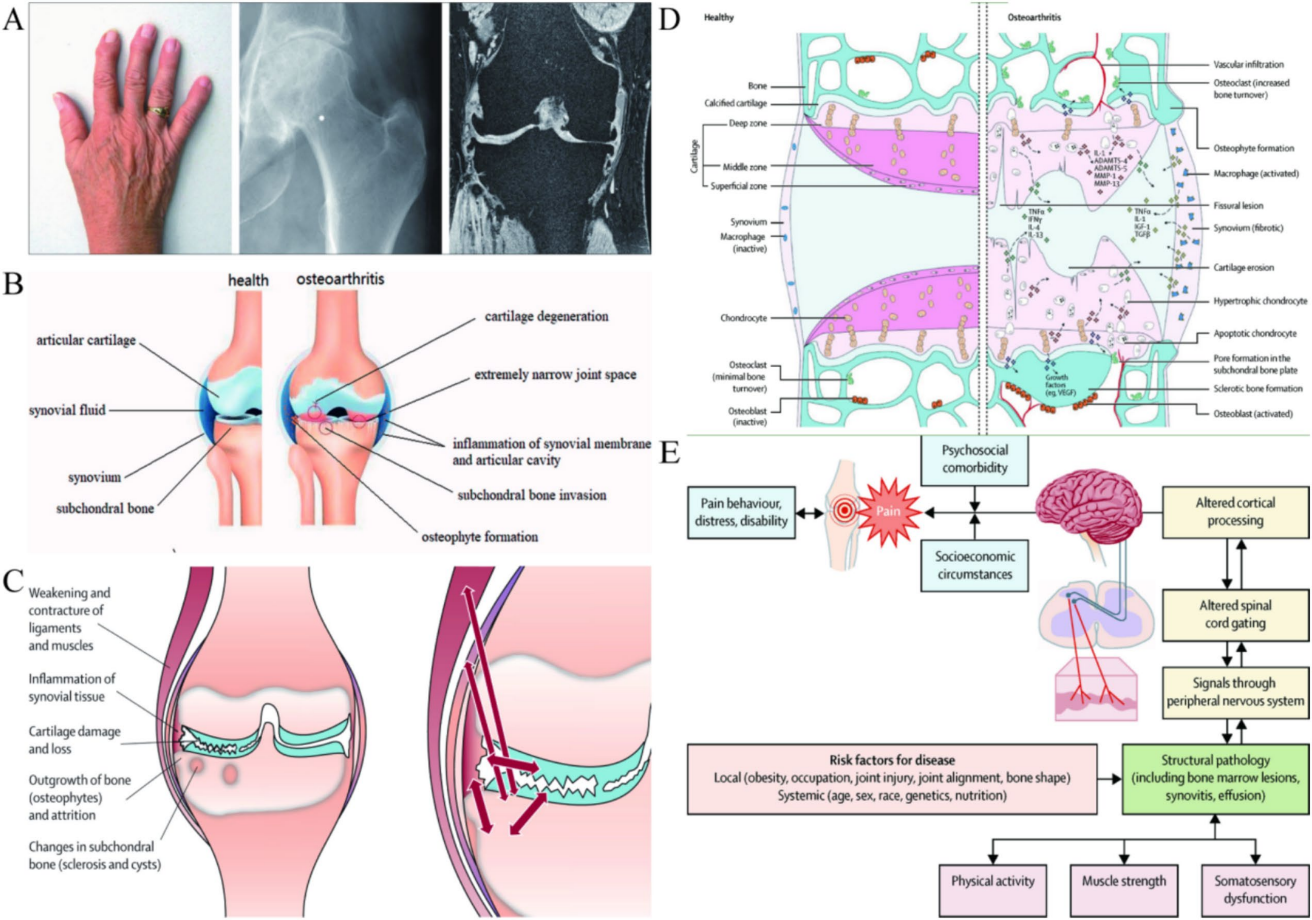
**(A)** Deformation of OA in the distal and proximal interphalangeal joints, plain radiograph of an osteoarthritic hip joint and MRI of an osteoarthritic knee. Reproduced from Bijlsma et al. (117). **(B)** Schematic representation of healthy knee joint structure and pathological changes of knee OA. Reproduced from Mao et al. (13). **(C)** Changes and interactions of different tissue structures in OA disease. Reproduced from Bijlsma et al. (117). **(D)** Signaling pathways and structural changes in the development of OA. Reproduced from Glyn-Jones et al. (89). **(E)** The cause of pain in OA within a biopsychosocial model. Reproduced from Hunter et al. (10).

OA, mainly characterized by degeneration of articular cartilage and osteophyte formation (Figure 1A), accompanied by degeneration of cartilage tissue, subchondral bone and synovium (Figure 1B), is a common chronic joint disease (1, 2). In addition to age, gender, and genetics, the causes of OA include abnormal increases in joint loading caused by factors such as obesity and occupation (3–5), disruption of mechanical balance due to fractures, meniscal injuries, and ligament tears (3) (Figure 1C), as well as damage to the infrapatellar fat pad (6, 7). With the aggravation of population aging and degeneration of the cartilage and meniscus, the incidence rate is increasing year by year, which not only brings great pain to patients themselves (Figure 1E), but also causes great social and economic burden (8). Patients often present with joint pain, swelling, and stiffness, eventually leading to chronic pain and physical disability (9, 10). However, the treatment of OA mainly focuses on relieving symptoms, divided into conservative treatment and surgical treatment. Conservative treatment mainly through physical therapy and drugs to alleviate the pain symptoms of OA, and surgical treatment in addition to joint replacement, other treatment is still difficult to prevent cartilage destruction (11, 12).

In the face of the huge medical demand, more and more research tries new treatment methods, including new drug development, intra-articular injection and the exploration of gene signal pathways (10) (Figure 1D). These studies mainly hope to prolong the effect of drugs, more accurate drug targeting and less treatment side effects (13). With the rapid development of biomaterials, we have focused on the great potential of nanoparticles (NPs) in the treatment of joint inflammation (14, 15). NPs refer to solid colloidal particles made of natural or synthetic polymer materials in the order of nanometer size (0.1–100 nm). The controllable size gives the feasibility of direct intra-articular injection (16–18). NPs are a promising cargo delivery system, which can bind drugs on the surface or substrate to protect drugs from enzymatic degradation, improve their permeability in cartilage matrix, and regulate the pharmacokinetics of drugs. And the NPs composed of biocompatible and biodegradable materials can achieve controlled and continuous drug release (19–21). This paper reviews the wide application of PLGA NPs in the treatment of OA in the past year, and explores the progress and challenges of PLGA in inflammatory arthritis based on cartilage degradation.

## 2 Overview of PLGA material

Poly(D,L-lactic-co-glycolic acid) (PLGA) is a biodegradable functional polymer organic compound composed of random polymerization of two monomers poly(lactic acid) (PLA) and poly (glycolic acid) (PGA) (22, 23). PLA degradation rate is slow, and PGA degradation rate is fast, so adjust the proportion of the two can regulate the mechanical properties and degradation time of polymer. We summarize the key data of recent studies cited in this review in Table 1, where the lactic acid/glycolic acid (LA/GA) ratio is shown to exert a significant regulatory effect on the degradation and drug release processes. At the same time, the polymer exhibits good biocompatibility, biodegradability, good encapsulation and film forming properties, and has been authorized by the food and drug administration for drug delivery system (24). At present, there are many preparation methods of PLGA particles, including: emulsion solvent volatilization method, microfluidic technology, spray drying technology, nanoprecipitation and phase separation, etc. (22, 25–27).

**Table 1:** PLGA degradation kinetics and drug release profiles of different LA/GA ratios.

Polymer	Composition	Degradation kinetics	Drug release profile	Reference
PLGA	LA:GA (50:50) + LA:GA (75:25)	1 month: moderate rupture	Burst release: 18–22% (24 h); Cumulative release: 78% (28d)	Sun et al. (35)
PLGA	LA:GA (50:50) (MW: 30–60 kDa)	28d: complete degradation	Burst release: 31.64% (24 h); Cumulative release: 95.45% (28d)	Wei et al. (37)
PLGA-F127	PLGA (50:50) (MW:100 kDa) + Pluronic F127	120d: complete degradation	Release onset: 60d; total duration: 4 months	Seon et al. (79)
PLGA	LA:GA (50:50)	28d: pore expansion	Burst release: 28.3% (24 h); Cumulative release: 85% (28d)	Zhu et al. (86)
PLGA	LA:GA (75:25) (MW: 20 kDa)	>34d: in cartilage	Burst release: <3%; Cartilage retention: >34d	Deng et al. (28)

The study demonstrates that varying LA/GA ratios in PLGA significantly influence particle degradation and drug release. When the LA/GA ratio was adjusted from 50:50 to 75:25, the degradation duration extended to 34 days with a 24-h burst release rate below 3%, which correlates with the increased LA content that slows particle degradation (28). In early-stage osteoarthritis (OA), where cartilage matrix integrity remains relatively high, the 75:25 LA/GA ratio enables sustained drug release through gradual penetration of the cartilage surface. However, in advanced OA characterized by cartilage matrix breakdown, the 50:50 PLGA formulation demonstrates accelerated degradation and rapid drug delivery through the compromised cartilage matrix.

## 3 The use of PLGA in OA

For the special anatomical structure of the knee joint, oral drugs are difficult to reach and form an effective local drug concentration, while the drugs injected in the arthrosis are quickly removed from the synovial fluid through the relevant lymphatic vessels and vasculature. To overcome these limitations, many NP-based drug delivery systems have been developed, such as PLGA-NPs delivering small-molecule drugs or endogenous growth factors (29, 30). The latest research have tried to explore the injectable *in situ* molding implants build intra-articular drug library. Sustainable release disease-modifying OA drug can improve OA. The sealing PLGA in original molding implant can stabilize for weeks and releasing drug can inhibit collagenase. However, such implants after formation exist certain cytotoxicity. Researchers may need to further modify to solve the problem of drug toxicity, but the method of implant treatment OA may be an innovative idea (31).

### 3.1 PLGA NPs delivering small-molecule drugs

Curcumin (CUR), as a natural polyphenolic compound, has potent assimilative, antioxidant, anti-inflammatory and anti-rheumatic properties. But the therapeutic efficiency of CUR is greatly limited due to its low water solubility and limited oral bioavailability (32, 33). One study on the preparation of PLGA NPs (CURNPs) containing curcumin for knee OA in rats, showed CURNPs inhibited the upregulation of several inflammatory factors, including interleukin-1β (IL-1β), tumor necrosis factor-α (TNF-α), and interleukin-6 (IL-6), and significantly retained type II collagen in articular cartilage. This is mainly achieved by inhibiting NF-κB pathway. Meanwhile, the radiographic and histological lesions of OA were significantly reduced (34). Sun et al. (35) prepared meloxicam-loaded PLGA microspheres (MLX-MS) by the emulsification-solvent evaporation method. An orthogonal test design was employed to optimize the formulation. Dynamic light scattering was used to measure the average particle size, which was controlled between 100 and 110 μm with a span of 0.5–0.6. The Fourier Transform Infrared Spectroscopy and X-ray Powder Diffraction were simultaneously utilized to confirm that there were no alterations in the drug during the encapsulation process. In the OA model of rats, various inflammatory factors including IL-1β, IL-6, and TNF-α were successfully inhibited, and the meloxicam exhibited a long-term sustained-release pattern. Meanwhile, compared to oral administration, local injection of MLX-MS significantly increased the elimination half-life and time to peak concentration in the plasma. Intra-articular injection of MLX-MS significantly reduced drug distribution in the gastrointestinal tract and allowed the drug for better penetration of the drug into the inflamed area. Zhu et al. (36) found that the small molecule drug salicin (SA) is important for the progression of OA, clarifying mechanisms by RNA sequencing, molecular docking and drug affinity-response target stability analysis *in vitro*. SA directly binds to IRE1α and occupies the IRE1α phosphorylation site, preventing IRE1α phosphorylation, and regulates IRE1α-mediated ER stress via IRE 1α-IκBα-p65. Injection of PLGA particles containing SA in the OA rats significantly improved OA progression. A large number of studies have shown that resveratrol (RSV) showed protective effects on articular cartilage through various mechanisms, including anti-inflammatory and anti-apoptosis, or regulation of signaling pathways or active factors. But resveratrol has the problems of poor chemical stability, poor water solubility and low bioavailability. Wei et al. (37) prepared RSV-PLGA NPs via the incorporation of RSV into PLGA. Using the good biocompatibility and stable drug release performance of PLGA, PLGA NPs significantly inhibited cartilage cell apoptosis and promoted glycosaminoglycan (GAG) synthesis and maintained continuous drug release for 35 days after a single injection in, supporting the superiority of intra-injection.

Previous studies have found that HDACi prevents the disruption of the extracellular matrix, and this destruction is induced by inflammatory factors (38–40). And, HDACi suppresses cartilage destruction and articular cartilage degeneration. So, Ye et al. (41) proposed that PLGA microcapsule delivered Chidamide to treat OA. Phenotype-associated genes of ECM were retained and increased in HDACi treated OA chondrocytes, and decreased expression of catabolism-related genes. The rat OA model later confirmed that Chidamide significantly reduced osteophyte formation on the medial side of the tibial plateau and effectively prevented the remodeling of the inferior tibial bone.

Rapamycin is also a well-known immunomodulator and antibiotic that has been used in a variety of clinical treatments and can delay the progression of OA in mouse models (42). Rapamycin induces cellular autophagy by inhibiting ribosomal protein S6 phosphorylation and affecting the mTOR signaling pathway, while reducing cellular senescence by increasing the levels of Nrf2 (42–44). Dhanabalan et al. (45) loaded rapamycin in PLGA particles, Rapamycin-loaded PLGA microparticles (RMPs) induce cellular autophagy in primary articular chondrocytes of OA patients, preventing senescence and continuous production of sulfated glycosaminoglycans. At the same time, the RMPs was retained in the mouse for up to 35 days, and PLGA particles with higher molecular weight can show longer residence time in the joint, significantly reducing the frequency of injection, which is beneficial to improve patient compliance in clinical practice.

Post-traumatic OA (PTOA) often begins with joint injuries including anterior cruciate ligament rupture, meniscus injury, and joint dislocation, along with progressive deterioration of the articular cartilage and subchondral bone (46). The particularity of the disease is that the inflammation is initiated at the molecular and cellular levels immediately after joint injury. Proinflammatory factors, including: IL-1β, IL-6, and TNFα, are rapidly induced (47). Subsequently the matrix-degrading enzymes such as matrix metalloproteinases, collagenase, and cathepsin are further induced (48). These factors cause irreversible damage to the surrounding tissues, eventually leading to OA (49).

**Figure 2:**
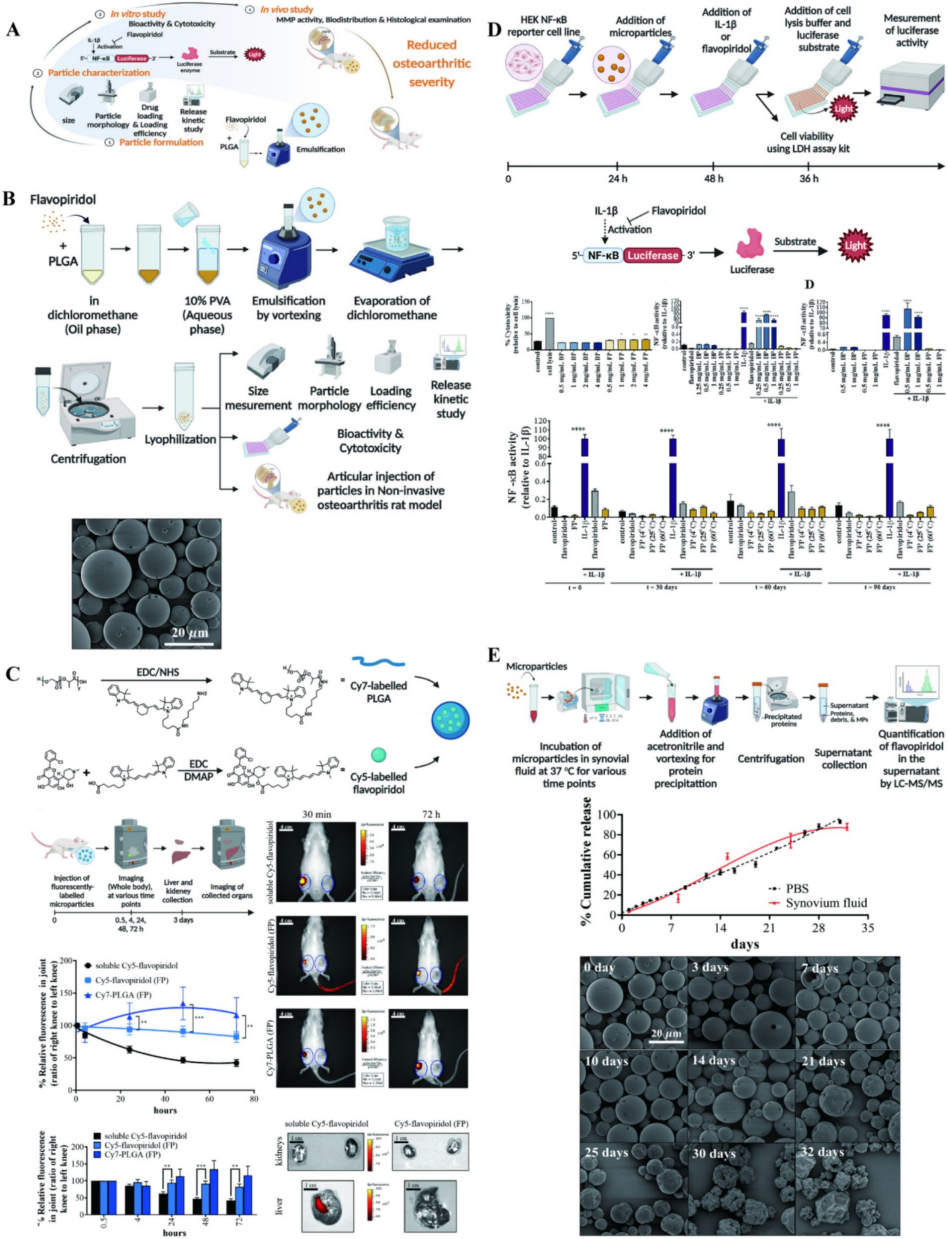
**(A)** The illustration on preparation and application of PLGA particle. **(B)** Preparation and characterization of flavopiridol-loaded PLGA particles. **(C)** Biodistribution of Flavopiridol-loaded microparticles (FPs) following intra-articular injection in rats. **(D)** NF-κB activity of FPs. **(E)** Release kinetic of FPs. Reproduced from Sangsuwan et al. (50).

The CDK 9 inhibitor Flavpiridol is a potent drug that prevents the acute inflammatory response and activation of catabolic pathways in cartilage, it has been shown that Flavpiridol suppressed the expression of inducible nitric oxide synthase and inflammatory mediator genes under proinflammatory stimuli. So, Sangsuwan et al. (50) prepared PLGA microspheres loaded with Flavpiridol to prevent PTOA by early reducing inflammation in rat knee joints, and found that the drug has less accumulation in the liver and kidney and is a potential PTOA candidate (Figure 2). Meanwhile, some studies applied PLGA microsphere loaded with Flavpiridol in the OA models of rabbit and horse. It more truly reflected the actual effect of the drug-loaded microsphere in large mammals, which also showed good slow-release lubrication, anti-inflammatory analgesia and articular cartilage protection (51, 52). Kim et al. (53) prepared NPs of two different materials for the delivery of rebamipide, demonstrating significant therapeutic effects both *in vivo* and *in vitro*. This study further showed the therapeutic potential of NP drug delivery systems within the unique anatomical structure of the joint cavity.

However, the hydrophobicity of drugs and molecular size significantly influence encapsulation efficiency in PLGA. As a hydrophobic polymer, PLGA demonstrates enhanced compatibility with hydrophobic drugs, thereby improving encapsulation efficiency. Additionally, drug molecular size plays a crucial role in PLGA loading and release kinetics: smaller molecular weight drugs diffuse more easily into PLGA but risk premature release during initial stages, while larger molecular weight drugs exhibit lower encapsulation rates due to steric hindrance effects, though they deliver slower release rates. These factors may warrant further investigation in future research.

### 3.2 PLGA NPs delivering the endogenous components

**Figure 3:**
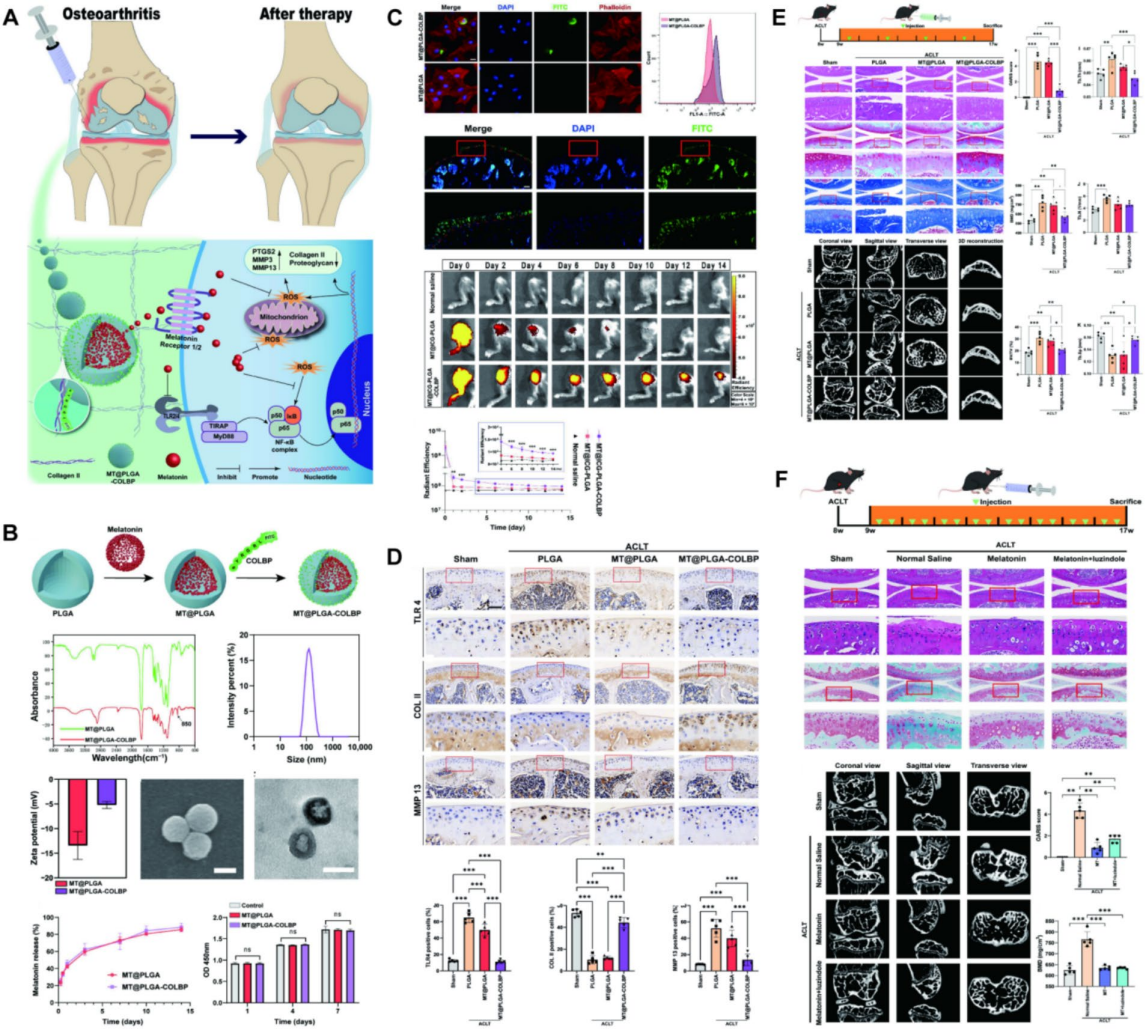
**(A)** The mechanism of melatonin-loaded nano-delivery system (MT@PLGA-COLBP) with cartilage-targeting effect for OA therapy. **(B)** Synthesis and characterization of MT@PLGA-COLBP NPs. **(C)** MT@PLGA-COLBP NPs target chondrocytes. **(D)** MT@PLGA-COLBP improves the protein expression of immune response and cartilage matrix *in vivo*. **(E)** Therapeutic effect of intra-articular injection of MT@PLGA-COLBP NPs on early OA mice. **(F)** Intra-articular injection of melatonin delays the development of OA in mice. Reproduced from Liang et al. (59).

Melatonin is an endogenous hormone secreted by the pineal gland, performing circadian regulation simultaneously with a potent antioxidant capacity. This hormone has great potential in osteoporosis, atherosclerosis, and diabetes, and has been reported for the treatment of OA (54–58). It is well known that ROS and TLR mediated cascade of inflammatory response OA has a critical role. Inhibition of the innate immune response and generation of reactive oxygen species are potential targets for treating OA. Liang et al. (59) packaged melatonin in PLGA by oil-in-water method and grafted type II collagenase in the surface. A nano-delivery system loaded with melatonin was prepared. Researchers evaluated the behavior of this system in cartilage and the therapeutic efficacy in mouse OA models, confirming that melatonin can protect chondrocytes by clearing ROS and inhibiting the TLR2/4-MyD88-NFκB pathway, and prevent degeneration of knee cartilage and remodeling of subchondral bone in early OA (Figure 3). The small molecule drugs and endogenous components delivered by PLGA and experimental studies on animal OA are outlined in Table 2.

**Table 2:** PLGA NPs delivering small-molecule drugs and endogenous components.

Carriers	Drugs/components	Damage	Induction	Animal	References
PLGA NPs	Curcumin	KOA	MIA injection	Rats	Curcumin NPs (34)
PLGA microspheres	Meloxicam	KOA	MIA injection	Rats	PLGA microspheres loaded with meloxicam (35)
PLGA	Salicin	KOA	ACLT	Rats	SA-loaded PLGA (36)
PLGA NPs	Resveratrol	KOA	DMM	Rats	PLGA resveratrol sustained-release NPs (37)
PLGA microcapsules	Chidamide	KOA	ACLT	Rats	Intra-articular Histone Deacetylase Inhibitor Microcarrier (41)
PLGA microparticles	Rapamycin	KOA	DMM	Mice	Intra-articular injection of rapamycin microparticles (45)
PLGA microspheres	Flavpiridol	KOA	ACLT	Rats	Intra-articular Injection of Flavopiridol-loaded Microparticles (50)
PLGA NPs	Melatonin	KOA	ACLT	Mice	Preparation of Melatonin-Loaded NPs (59)
PLGA microspheres	Nanofat	KOA	DMM	Rats	Nanofat functionalized injectable super-lubricating microfluidic microspheres (64)
PLGA NPs	P16INK4A-siRNA	KOA	PMMx	Mice	p16INK4a-siRNA NPs (68)

PLGA, Poly(D,L-lactic-co-glycolic acid); NPs, Nanoparticles; KOA, Knee Osteoarthritis; MIA, Monosodium Iodoacetate; ACLT, Anterior Cruciate Ligament Transection; DMM, Destabilization of Medial Meniscus; PMMx, Partial Medial Meniscectomy.

**Figure 4:**
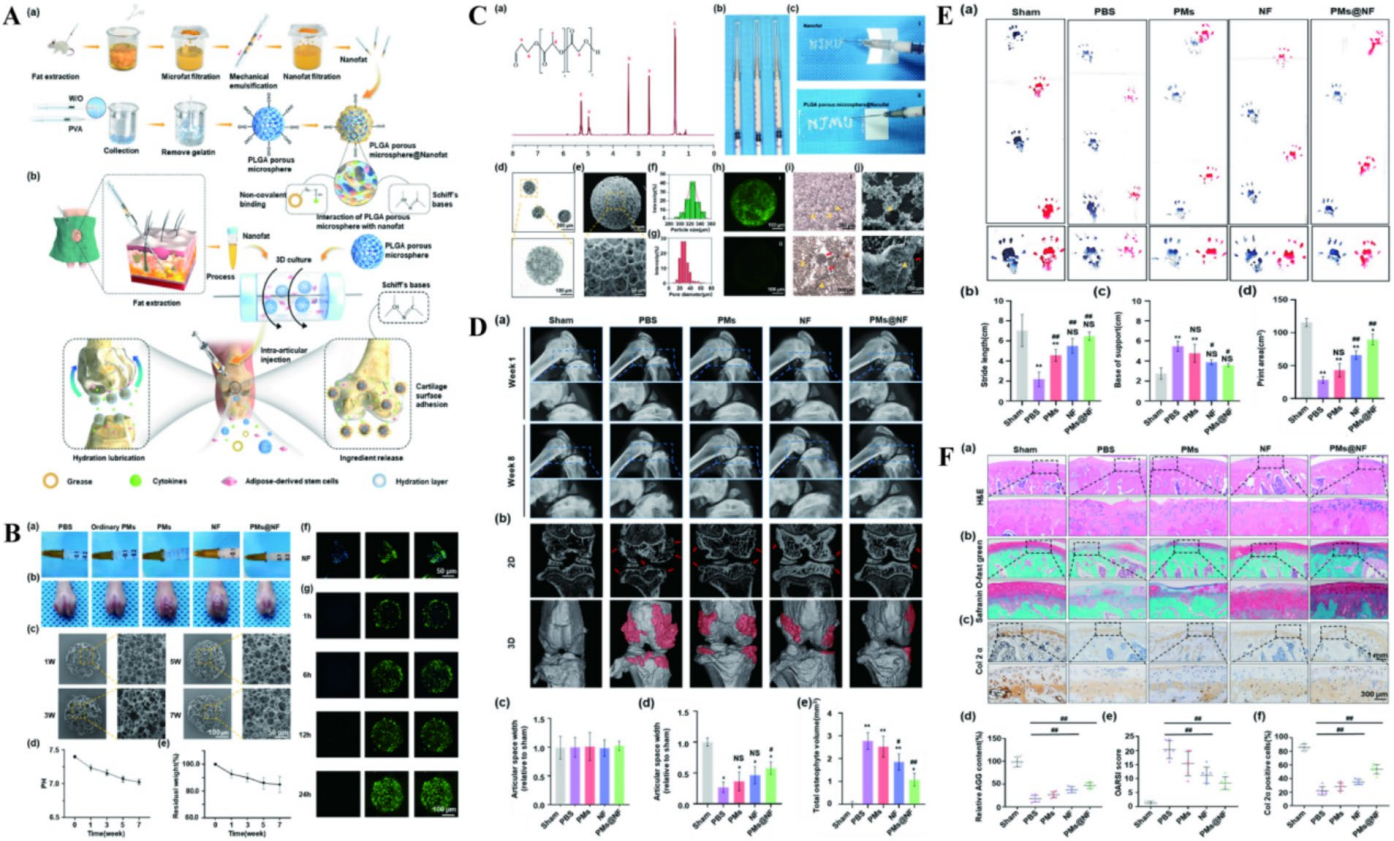
**(A)** The illustration preparation of NF, PMs and PMs@NF and the clinical application of multifunctional microfluidic PMs@NF. **(B)** Intra articular injection, *in vitro* degradation analysis and Calcein AM/DAPI staining of PMs@NF. **(C)** Characterization of NF functionalized injectable super-lubricating microfluidic PMs. **(D)** The performance of super-lubricating PMs@NF treat OA model in vivo. **(E)** Footprints collection of rats 8 weeks postoperatively. **(F)** Super-lubricating PMs@NF protects cartilage from invariance. Reproduced from Han et al. (64).

Nanofat (NF) is an injectable sticky extract rich in lipids, growth factors and stem cells, and NF has been successfully used in scar repair, vascular regeneration and cartilage defect repair (60–62). However, it is difficult to apply to precise transplantation due to the low mobility and low biological activity of NF (63). To overcome these limitations, Han et al. (64) prepared three-dimensional PLGA porous microspheres using microfluidic techniques. Combining NF into PLGA porous microspheres (PMs) through Schiff base condensation and noncovalent binding (PMs@NF). The construct loaded a large amount of biologically active NF, increased the local cytokine concentration secreted by stem cells. At the same time, it has targeted adhesion to the surface of cartilage, which can achieve accurate delivery. The structure not only strengthened the surface lubrication of articular cartilage, but also increased the expression of cartilage synthetic substances (Figure 4). PMs@NF downregulated the expression of genes involved in cartilage catabolic enzymes, inflammation and pain, significantly reducing osteophyte formation in arthritic rats. NF also activates the intracellular PI3K/Akt signaling pathway, promotes the proliferation and matrix synthesis of chondrocytes, and ultimately improves the progression of OA. Cellular senescence is an important factor in the pathogenesis of OA. Chondrocytes produce a senescence-related secretory phenotype (SASP), including inflammatory cytokines and matrix remodeling regulating metalloproteinases, causing chronic inflammation and ultimately leading to OA (65, 66). The cell cycle inhibitor P16INK4A protein has an important role in cellular senescence. Chondrocytes positive for the cell cycle inhibitor P16INK4A protein will secrete large amounts of inflammatory cytokines. These inflammatory factors often accumulate in the tissues and promote tissue degeneration through persistent chronic inflammation and extracellular matrix remodeling (67). However, the role of cell cycle inhibitor P16INK4A protein in OA and its inhibitory effect have not been defined, Park et al. (68) found that a marked increase in the cell cycle inhibitor P16INK4A protein in synovial and articular chondrocytes from OA patients. They used PLGA NPs to deliver the cell cycle inhibitor P16INK4A protein-siRNA. This NPs can significantly reduce the levels of TNF-α, IL-1β, and IL-6. In the mouse model of OA, NPs mainly focused on the synovial membrane, preferentially reducing the cell cycle inhibitor P16INK4A protein in synovial cells, reducing synovial inflammation and relieving joint pain. The authors were combined with previous studies (69, 70), speculating that fibroblasts in the synovial membrane may be new and exciting targets for OA therapy, promise as a potential drug to slow the progression of OA.

### 3.3 PLGA-based hybrid systems for combinatorial therapy

Ketorolac, as a non-steroidal anti-inflammatory drug for OA, is injected into the joint to inhibit the synthesis of prostaglandins and exerts antipyretic, analgesic and anti-inflammatory effects (71, 72). However, the drug has a short half-life *in vivo* and requires frequent injection, causing poor patient compliance. So, the researchers prepared a variety of ketorolac particles by different types of polymer materials to increase the slow-release properties of the drug, including: polymethacrylate, ethyl cellulose, chitosan, polycaprolactone, PLGA and the blend of two polymers, such as chitosan/gelatin (71, 73). Through modulation of two polymers, polymer blends can have customized drug release and improved physicochemical properties. In the study conducted by Wongrakpanich et al. (74), using two emulsification techniques, probe ultrasonication (PS) and high-speed agitation (HSS), prepared PLGA and PLA mixed polymer particles by water-in-oil-in-water (w/o/w) double emulsion solvent evaporation. Two emulsification techniques prepared drug-loaded microspheres with different particle size ranges. These microspheres delivered ketorolac with hyaluronic acid to treat OA. Found that the PS particles exhibited higher drug release within 24 h, whereas the HSS particles exhibited sustained release for more than 35 days. The combination of the two preparations as an alternative to OA treatment required only a monthly use.

Currently for OA in chronic inflammatory diseases and rheumatoid arthritis in autoimmune diseases, injecting corticosteroids into the joint is still an effective way to control pain and reduce inflammatory responses (75, 76). Triamcinolone sustained-release injections based on PLGA microsphere have been developed, however this injection has a stronger toxic effect on cells than dexamethasone at high doses (77, 78). Thus, Seon et al. (79) prepared self-assembled microspheres for microprecipitation reactions, loaded with dexamethasone, using PLGA and Pluronic®F-127 (F127). The in-situ implant within the joint cavity is formed by utilizing the characteristic of F127 solution to transition from sol to gel when it is above the lower critical solution temperature. By comparing the drug release between PLGA-F127 microspheres and PLGA-only microspheres, it was found that the in-situ implant did not exhibit initial burst release, but started releasing the drug after being retained for 60 days. The entire drug release time can reach up to 4 months. Compared to other drug delivery systems, PLGA-F127-MS demonstrates potential as a novel sustained-release drug delivery system.

In OA studies, abnormal subchondral bone remodeling is the main phenotypic feature of early stage of OA. Osteoclast activation, and locally elevated transforming growth factor (TGF)-β1 exacerbates early subchondral bone loss. They induce vascularization and hypomineralization in osteoblasts, leading to sclerosis during OA progression (80–82). TGF-β may be a potential therapeutic target for treating OA. Pirfenidone (PFD) is a pyridine-like small-molecule TGFβ1-3 inhibitor, which can specifically inhibit the TGFβ signaling pathway to exert anti-fibrotic and anti-inflammatory effects. This drug has been used clinically for treating various pathological fibrosis including pulmonary fibrosis and liver fibrosis (83). Some studies have demonstrated the effectiveness of oral PFD for OA, but long-term, high-dose oral PFD caused a wide range of side effects (84, 85). Zhu et al. (86) controlled the local concentration of PFD by preparing PLGA microsphere loaded with PFD. This microsphere combined with hyaluronic acid solution allowed sustained release PFD in the joint cavity, preventing subchondral bone loss in early OA and subchondral bone sclerosis in late OA. Meanwhile, this combination alleviated synovial inflammation and pain-related behavioral changes and achieved the disease-modifying effect of early OA.

Between the formation and resorption of bone and cartilage, there are a variety of cells that can influence OA progression, including mesenchymal stem cells (MSCs), chondrocytes, and osteoclasts (87, 88). Magnesium, as a skeletal system element, has an important role in the maintenance of skeletal and cartilage health processes. It has been shown to effectively stabilize nucleic acids and enzymes to exert therapeutic effects on a variety of cells in OA progression (89). The latest research utilized magnesium treatment to chondrocytes in the simulate environment of OA. It was found that AKT phosphorylation was activated, and the apoptotic markers cleaved calpain I and BAX/BCL2 were significantly reduced at the protein level, resulting in a significant decrease in the rate of cell apoptosis. Meanwhile, the phosphorylation level of AKT in osteoclasts was significantly decreased, and transcriptome sequencing analysis revealed significant changes in the PI3K/Akt pathway. Researchers have prepared stearic acid (SA) modified PLGA microspheres loaded with nano-magnesium oxide—MgO&SA@PLGA (90). This microsphere provided a suitable concentration of Mg^2^, which not only promoted MSC proliferation and chondrogenic differentiation but also effectively inhibited osteogenic differentiation. The treatment demonstrated significant relief effects in the rat. This study provided further support for the use of magnesium in the treatment of OA-related issues in cartilage and subchondral bone by activating the AKT signaling pathway.

**Figure 5:**
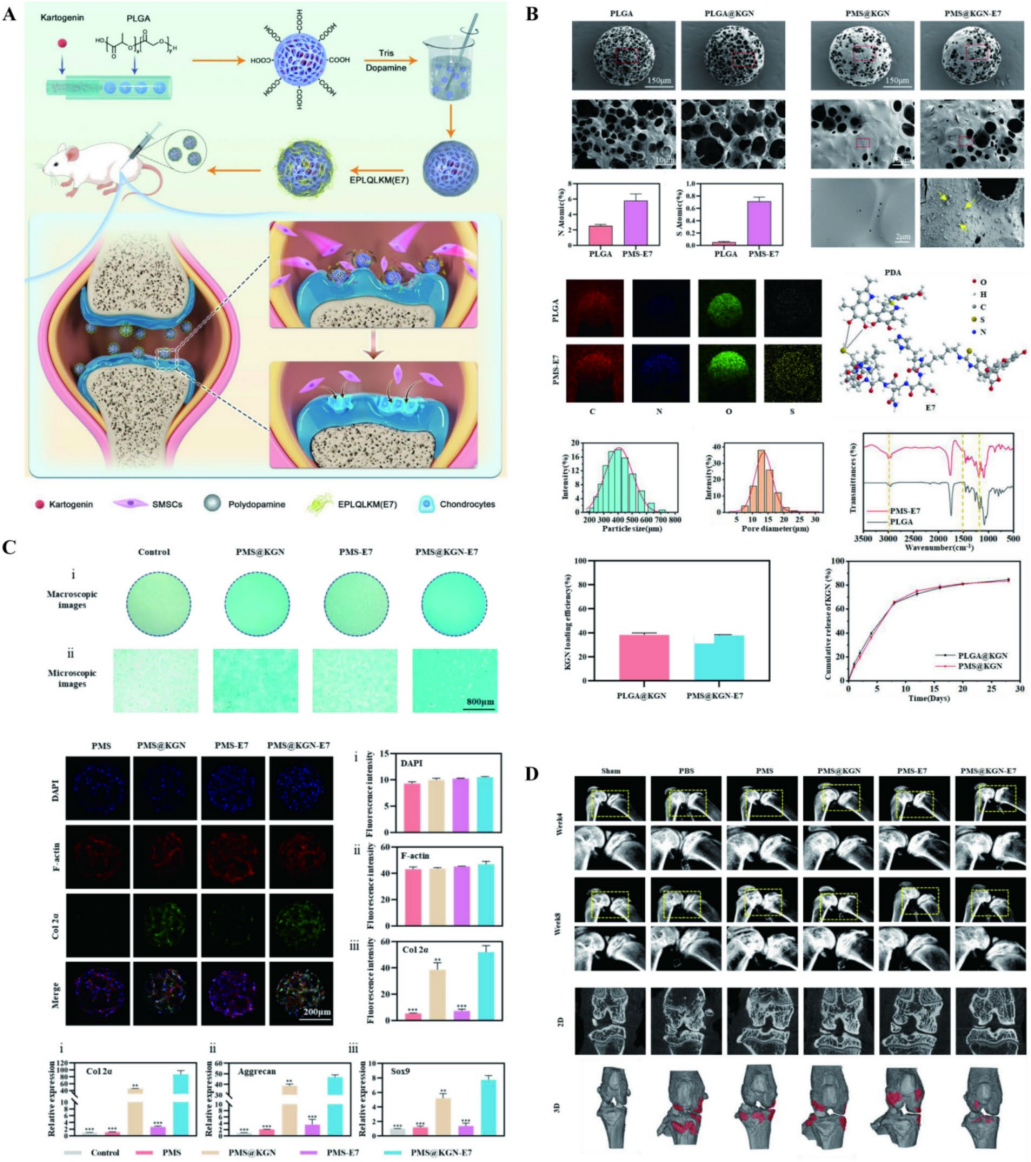
**(A)** The illustration on preparation and application of multifunctional cartilage repair microspheres. **(B)** Morphological and characterization of microspheres. **(C)** Microspheres promote chondrogenic differentiation. **(D)** Microspheres treatment and morphological assessment. Reproduced from Zhang et al. (95).

Kartogenin (KGN) is a hydrophobic small molecule drug that can significantly promote chondrogenic differentiation of bone marrow mesenchymal stem cells and can induce cartilage regeneration in OA (91–94). However, KGN, as a small molecule, has poor water solubility, and its therapeutic effect is not efficient when used alone. Therefore, the use of nanocarriers may be beneficial for drug therapy and intracellular drug delivery. In the early stage of OA, irregular partial defects occur in articular cartilage, causing progressive destruction of OA. Zhang et al. (95) prepared PLGA microspheres uniformly loaded with KGN using microfluidic technology (KGN@PLGA NP). Subsequently, the PLGA microspheres were modified with dopamine to form a dopamine coating KGN@PLGA NP. Finally, the E7 recruiting peptide was non-covalently bound to the KGN@PLGA NP to prepare an injectable multifunctional PLGA microsphere for repairing partial defects in cartilage. The PDA coating could enhance the adhesion ability of PLGA microspheres, while the E7 peptide could recruit endogenous stem cells. After being injected into the joint cavity, the multifunctional microspheres could adhere to the damaged cartilage matrix and release the E7 peptide to recruit stem cells to the lesion area. With the degradation of PLGA, the release of KGN induced osteogenic differentiation of stem cells. Ultimately, the cartilage surface in the treatment group became smooth and the GAG content returned to normal, indicating the microspheres could promote the repair of cartilage injuries. New research has shown that the use of phenol-rich PDA can effectively scavenge ROS, reduce acute inflammatory responses, and inhibit the enhancement of osteoclasts (Figure 5). PLGA/polydopamine-based core/shell NPs loaded with KGN can significantly induce cartilage synthesis *in vitro*, and simultaneously effectively protect cartilage and subchondral bone in OA models of rats (96). Bai et al. (97) utilized nanomaterials as carriers for stem cell expansion, attempting to treat OA through stem cell tissue engineering. Firstly, PLGA porous microspheres loaded with KGN were prepared by an emulsification method. These microspheres were then anchored with chitosan (CS) using an amidation reaction to prepare PLGA-CS@KGN porous microspheres, which were subsequently cocultured with mesenchymal stem cells. It was found that the system has the ability to carry high cell densities of 1 × 10^4^ mm^−3^ and can protect MSCs by controlling their release, migration, and proliferation in an inflammatory microenvironment. This system also provided prospects for the treatment of OA through stem cell tissue engineering.

Besides delivering drugs, the use of NPs to deliver endogenous components for regulating the progression of OA is also a promising therapeutic approach. MSCs play a leading role in tissue engineering and regeneration. They primarily regulate the local microenvironment by secreting bioactive molecules and possess multidirectional differentiation capabilities, enabling them to renew and differentiate into various lineage cells such as fat, bone, cartilage, tendons, and skin (98). MSCs have been widely used in preclinical research. However, the application of conditioned media during cell culture *in vitro* still requires extensive cell isolation and maintenance, and there are risks of spontaneous behavior and property changes, including cell contamination, infection transmission, and malignant tumors (98–100). Meanwhile, the main executors of therapeutic effects are paracrine factors secreted by stem cells in tissue engineering regeneration. Therefore, Shah et al. (101) selected growth factors of great significance to chondrogenesis, including insulin-like growth factor, TGF-β1, fibroblast growth factor-18, and human growth hormone. They encapsulated the recombinant proteins of these growth factors in PLGA to construct a synthetic artificial stem cell system. The system constructed exhibited significant anti-inflammatory and chondroprotective effects *in vitro*, and alleviated cartilage degeneration and improved the biomechanical properties of articular cartilage *in vivo*, verifying the possibility of a new therapeutic strategy for OA.

MicroRNA (miRNA) is a class of non-coding single-stranded RNA molecules, approximately 22 nucleotides in length, encoded by endogenous genes and involved in post-transcriptional gene expression regulation in both plants and animals (102, 103). Several important microRNAs have been identified to play crucial roles in the progression of OA, including as regulators of pro-inflammatory and matrix-degrading mediators (102). Among them, miR-140 is specifically expressed in human articular chondrocytes, and dysregulation of miR-140 can promote chondrocyte inflammation and lead to degenerative lesions. Especially, downregulation of miR-140 can cause excessive activation of ADAMTS-5 signaling, leading to the loss of proteoglycan and type II collagen in chondrocytes (104, 105). Zhao et al. (106) aimed to slow the progression of OA by delivering recombinant miR-140. Firstly, a cartilage-targeting peptide (CAP)-modified poly(vinyl amine) (PVAm)-PLGA copolymer was prepared. And then it was formed into spherical NPs with r-miR-140 (CPPNPs). The introduction of PLGA significantly improved the mechanical properties and stability of CPPNPs. Meanwhile, CAP endowed the NPs with cartilage targeting ability, enabling CPPNPs to exhibit significant permeability and accumulation in cartilage and subchondral bone, thereby overcoming the two major biological barriers of cartilage: avascularity and high ECM density. In the mouse model, CPPNPs treatment significantly reduced cartilage degeneration and synovial inflammation, providing a new foundation for delivering RNA to treat OA by overcoming cartilage barriers.

However, with the progression of OA, the increase in cartilage damage leads to the gradual loss of cartilage components, weakening the abilities of passive targeting and active targeting of single ligands. Therefore, compared to targeting a single component of cartilage, a delivery system with multiple affinity peptide ligands is more capable of overcoming the difficulties of the extracellular matrix barrier of chondrocytes and cartilage targeting (107–109). Deng et al. (28) inspired by chondrocyte-matrix interactions, utilized naturally derived chondrocyte membranes (CMs) as a mean of enhancing the specificity and binding capacity of drug delivery. Firstly, PLGA particles loaded with the Wnt pathway inhibitor adavivint were prepared, and then naturally derived chondrocyte membranes were coated on the surface of the PLGA particles for modification. It was found that NPs coated with chondrocyte membranes could be preferentially taken up by chondrocytes, mainly through membrane protein-targeted recognition and clathrin-mediated endocytosis, as well as micropinocytosis. Meanwhile, these CM-NPs could overcome the extracellular matrix barrier of chondrocytes, penetrate into the cartilage matrix, and remain there for over 34 days, effectively protecting articular cartilage and alleviating the progression of OA. The combination of natural cell membranes with synthetic NPs could disguise the NPs as endogenous cells, effectively avoiding clearance by immune cells and extending the duration of treatment. The cell membrane on the surface can also exert corresponding biological effects, making it an OA treatment strategy with almost no side effects (110). Previous studies have shown that MSCs can promote cartilage proliferation and may restart the chondrocyte cycle. Human synovial CD90-positive MSCs may be involved in cartilage repair in OA (111, 112). Li et al. (113) utilized cytochalasin B to stimulate CD90 + MSCs to secrete microvesicles (CD90@MV) and prepared PLGA NPs encapsulated with triamcinolone acetonide (TA) within these microvesicles (T-CD90@NP). It was found that the membrane proteins of CD90@MV were similar to those of CD90 + MSCs, and their bioactivity was comparable to that of CD90 + MSCs in inducing cartilage proliferation. T-CD90@NP demonstrated significant cartilage repair and anti-inflammatory capabilities in OA models of rats and rabbits, capable of inducing cartilage to restart the cell cycle and reducing chondrocyte apoptosis. Bioinformatics analysis and mRNA sequencing confirmed that T-CD90@NP mainly reduced cell apoptosis through the FOXO pathway and regulated inflammation by affecting M2 macrophage polarization through IL-10. The study of PLGA with other materials co-delivering drugs for OA model treatment is outlined in Table 3.

**Table 3:** PLGA with other materials.

Carriers	Components	Preparation	References
PLGA and PLA	Ketorolac	The w/o/w double emulsification solvent evaporation	Ketorolac-Loaded PLGA-/PLA-Based Microparticles (74)
PLGA and Pluronic®F-127	Dexamethasone	Microemulsion	Self-Assembled PLGA-Pluronic F127 Microsphere (79)
PLGA microparticles and HA solution	PFD	The o/w emulsification solvent evaporation	Intra-articular sustained-release of PFD (86)
SA modified PLGA microspheres	MgO	N/a	Engineered MgO NPs (90)
PLGA/PDA-PEG-E7	KGN	Microfluidic technology	Intra-Articular Injection of PLGA/Polydopamine Core−Shell NP (96)
PLGA-CS	KGN/BMSCs	Emulsification solvent evaporation	Stem cells expansion vector via bio-adhesive porous microspheres (97)
PLGA	IGF1, TGF-β1, FGF-18, and HGH	The w/o/w double emulsification solvent evaporation	The synthetic artificial stem cell system (101)
CAP-PVAm-PLGA	R-miR-140	N/a	Co-polymer carrier with dual advantages of cartilage-penetrating and targeting (106)
CM-NPs	Adavivint	Extrusion	Chondrocyte membrane–coated NPs (28)
CD90@NP	Triamcinolone acetonide	Water bath ultrasound	Triamcinolone acetonide-loaded NPs (113)

PLGA, Poly(D,L-lactic-co-glycolic acid); PFD, Pirfenidone; KGN, Kartogenin.

## 4 Challenges and prospects

Due to its excellent biocompatibility and biodegradability, PLGA has proven to be an excellent carrier for controlled drug, peptide, and protein delivery (21, 114). Many studies utilizing PLGA particles for OA treatment have achieved significant results *in vivo* or *in vitro* experiments (115, 116). PLGA particles show great potential in preclinical models of OA. However, the systems and methods used for evaluation are mainly based on lower mammalian models such as mice and rabbits (29).

There are still many shortcomings in the research of PLGA delivery system. Rodent knee joints feature smaller volumes and simpler anatomical structures, enabling rapid and uniform drug distribution within the cavity. In contrast, human knee joint cavities are more complex and contain larger volumes, resulting in significant variations in drug delivery patterns. Additionally, the significantly thicker articular cartilage in humans imposes higher penetration requirements for drug particles, potentially necessitating adjustments to the LA/GA ratio or the introduction of targeted delivery strategies. Furthermore, rodents exhibit denser vascular and lymphatic networks in synovial tissues, coupled with faster clearance rates within the joint cavity, presenting new challenges for particle metabolism and product accumulation in human joints. To address these challenges, experimental validation using large animal models serves as a crucial translational bridge.

Given the repeated drug injections in osteoarthritis patients, the degradation of PLGA into lactic acid and glycolic acid within joints may alter the microenvironment. Therefore, we propose regulating the degradation rate of PLGA to ensure the production and rate of metabolites remain within the metabolic tolerance threshold of the joint microenvironment. Additionally, repeated injections should not activate immune cells. Future studies could further mitigate potential effects by introducing neutralizing components or extending the degradation cycle.

The rapid release of drugs from PLGA carriers primarily results from free drug dissolution. Surface modification strategies are employed to regulate drug distribution through physical or chemical modifications. These include PDA surface coatings that reduce direct drug exposure and bind to drugs via π–π bonds, as well as chondrokinin peptide modifications that form a hydration layer on the carrier surface to slow drug diffusion into synovial fluid. Another method involves multi-layer coating techniques: HA coatings create gel-like barriers while chitosan/sodium alginate coatings generate electrostatic attraction. By modifying the composition and main chain structure of PLGA copolymers, drug release kinetics can be optimized at the source. For instance, blending PLGA with Pluronic F127/PCL block copolymers enables sustained drug delivery.

Conventional single-target PLGA delivery systems struggle to address the complex pathological process of OA involving cartilage degradation, synovial inflammation, and subchondral bone remodeling. Multi-target synergistic delivery, leveraging the controlled release properties of PLGA carriers, enables precise regulation of active components targeting OA tissue lesions. The next-generation PLGA delivery systems could integrate anti-inflammatory drugs with RNA or growth factors to achieve coordinated modulation of synovial cartilage or subchondral bone. Long-term validation in large animal models would better align with the clinical manifestations of OA.

Therefore, we need to establish a drug prioritization framework centered on disease-modifying effects, emphasizing therapeutic interventions that go beyond symptom relief. This approach will prove valuable for OA treatment. The strategy comprises three key components: (1) PLGA composite systems targeting cartilage repair and multi-pathological mechanisms (including inflammation suppression, matrix degradation inhibition, and subchondral bone protection), focusing on disease-modifying effects; (2) PLGA gene delivery systems targeting core signaling pathways of cellular senescence and apoptosis; (3) PLGA small-molecule delivery systems providing long-term anti-inflammatory action and mild cartilage protection, primarily aimed at symptom management.

Critical barriers remain to further advance the clinical translation of this material, with two core bottlenecks: large-scale Good Manufacturing Practice (GMP) production of the drug delivery system and long-term safety and efficacy assessment.

First, there is a significant gap between small-scale laboratory preparation and large-scale GMP production. Current research mainly relies on small-batch fabrication techniques, such as microfluidics or emulsification-solvent evaporation. These methods exhibit excellent performance in particle size control and drug loading efficiency. However, during GMP scale-up production, insufficient homogenization pressure or improper surfactant concentration can compromise the size stability of particles. Meanwhile, subtle variations in monomer purity and polymerization conditions across production batches may alter the *in vivo* drug release cycle of the system, thereby impairing therapeutic efficacy. Second, the innovative composite systems developed in many studies require multi-step modification and detection, which can be precisely controlled in small-scale laboratory research. In contrast, during large-scale GMP production, each modification step demands strict quality control and aseptic processing—including verification of particle targeting and functionality, as well as aseptic handling and storage of particles. This significantly increases production costs and process complexity.

At present, the hybrid system has key value in application prospect. For instance, the core of GMP compliance for PLGA/PDA hybrid systems lies in the controllable and stable PDA coating process. Selecting microfluidic technology to control the coefficient of variation in PDA coating thickness may ensure batch stability. In collagen-mixed systems, standardization of collagen raw materials and compatibility of composite processes require consideration. Commercially available recombinant collagen could be adopted to avoid batch variations from animal sources, while developing sterilization processes that preserve collagen activity. For cartilage cell membrane hybrid systems, limitations mainly stem from cell membrane sourcing and standardized extraction/quality control systems. Future efforts might involve establishing standardized cell banks, using flow cytometry for membrane protein quality control, and developing long-term storage solutions for cartilage membranes under GMP conditions. The GMP transformation of hybrid systems should focus on three core aspects: raw materials, processes, and quality control. By controlling batch variations in raw materials and reducing manual operations, clinical applications can be progressively achieved.

Meanwhile, conducting stability testing and long-term storage studies on PLGA delivery systems prior to clinical application holds translational value. The testing focuses on the physical, chemical, and biological stability of the delivery system to ensure structural integrity of the PLGA carrier and stable drug release kinetics. It also verifies that the drug degradation and its resulting PLGA decomposition products will not affect the microenvironment of the joint cavity. Ultimately, validation must confirm that the PLGA delivery system maintains activity *in vivo* and remains stable even after prolonged storage, thereby meeting clinical requirements.

Finally, research on the long-term *in vivo* safety and efficacy of this drug delivery system remains inadequate. Current animal studies have short evaluation periods and primarily use young individuals, whereas clinical OA patients are mainly elderly, and OA treatment may last for several years. Consequently, clinical OA treatment may require repeated intra-articular injections. Long-term injection and degradation of PLGA may lead to changes in the pH of synovial fluid, and long-term accumulation of particles in articular cartilage or subchondral bone may exacerbate metabolic changes in osteocytes. Additionally, elderly individuals often have impaired renal and hepatic function, resulting in reduced clearance rates of degradation products and a subsequent increase in the risk of systemic toxicity. Although PLGA-based composite systems with other materials may offer advantages in targeting and functionality for OA treatment, the immunogenic risks associated with long-term *in vivo* exposure to these components also warrant attention.

The elderly and metabolically impaired populations constitute the primary group affected by OA. The joint microenvironment in these groups shows significant differences compared to young, healthy animals. Therefore, evaluating the repair efficacy and safety of OA animal models is essential, while assessing the inflammatory regulation and microenvironment compatibility of PLGA formulations in metabolically impaired models holds practical value. We advocate establishing comparative evaluation systems between aged/metabolically impaired animals and their younger counterparts in future research. This approach is crucial for advancing the clinical translation of such systems.

Although there are still many problems to be solved in the treatment of human OA using NPs, we have seen that NPs exhibit good effects in drug delivery, tissue engineering, and gene therapy, providing new ideas for disease treatment. The PLGA particles discussed in this review also demonstrate great potential in OA treatment. Their unique properties make them powerful tools for new therapeutic methods and treatments, including loading small molecule drugs, recombinant proteins, and gene delivery, as well as hybrid modifications with different materials or endogenous biological components. Future research should align with the development of PLGA systems, including the development of GMP compliant manufacturing technologies, the establishment of unified quality testing standards for finished products, and the conduct of long-term safety and efficacy studies in aged animal models.

**Figure 6:**
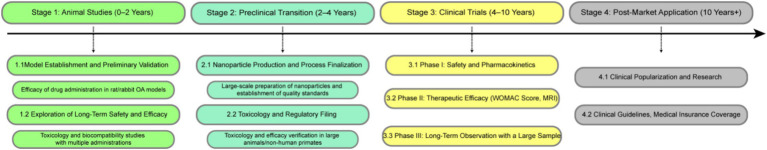
Proposed clinical research roadmap of PLGA delivery systems for OA therapy.

The combination of NPs with gene therapy may be a new direction for OA therapy. Such as the binding of exosomes to PLGA NPs, which can carry an siRNA targeting key genes of OA. Exosomes also have good biocompatibility and targeting properties. Targeted treatment of cell membrane camouflage is also of concern, which can not only deceive immune cells but also target cell and tissue repair. NPs combined with immune regulation also have great potential, inhibiting the inflammatory response to provide a favorable microenvironment for cartilage repair. In the future, multi-dimensional integration of these mechanisms may be needed to further prevent the development of OA. Therefore, we present a perspective on the clinical translation of this drug delivery system (Figure 6).

Beyond these aspects, combined therapies demonstrate high efficacy in OA clinical practice. Non-pharmacological interventions enhance joint function and modulate the local microenvironment, thereby supporting the therapeutic effects of PLGA delivery systems. Physical therapy regulates blood flow, tissue permeability, and cellular activity in the joint area through physical energy modulation, which enhances the efficacy of PLGA-delivered medications. In conservative OA treatment, strength training improves joint stability and cartilage nutrition supply, while PLGA’s sustained delivery provides continuous protection for cartilage during physical activity. Mechanical decompression devices reduce joint load, helping minimize inflammation and cartilage damage caused by excessive wear, creating a critical window for cartilage repair. Therefore, future research should build upon the PLGA studies outlined in this review and further validate the long-term effectiveness of combined therapies in large animal models.

We should establish a comprehensive evaluation system for standardized preclinical protocols, including dose adjustments tailored to PLGA’s inherent properties and animal models across small animals, large animals, and humans. The trial duration should be structured into distinct disease progression phases: acute phase, chronic phase, and long-term follow-up. Simultaneously, multiple biomarkers within the body must undergo safety and efficacy testing, encompassing both safety biomarkers and therapeutic biomarkers, along with imaging and functional metrics. These established standards may provide new insights for future research.

We believe that with the advancements in material engineering and drug delivery technology, the PLGA NP delivery system will receive more attention and exploration in scientific research, further promoting clinical studies on PLGA NPs in OA treatment. Therefore, the summary of this review is beneficial for readers to understand the latest research findings and corresponding challenges of PLGA NPs used in OA treatment.
